# Current trends in cacti drying processes and their effects on cellulose and mucilage from two Colombian cactus species

**DOI:** 10.1016/j.heliyon.2022.e12618

**Published:** 2022-12-24

**Authors:** Oscar J. Medina, William Patarroyo, Lucia M. Moreno

**Affiliations:** Grupo de Investigación en Química y Tecnología de Alimentos, Universidad Pedagógica y Tecnológica de Colombia, Colombia

**Keywords:** *Opuntia ficus-Indica*, *Austrocylindropuntia cylindrica*, Refractance window, Conventional oven, Drying kinetics

## Abstract

The effect of temperature and drying technologies on mucilage and cellulose (obtained by the microwave-assisted extraction technique, MAE) from *Opuntia ficus-indica* (OFI) and *Austrocylindropuntia cylindrica* (CC) was determined using a conventional oven (CO) and Refractive Window (RW). Mathematical modeling was performed from drying kinetics data using the Lewis, Henderson-Pabis, Page, and Logarithmic models. Activation Energy (Ea) and Diffusivity (D) were also determined. The model with the best fit was the logarithmic one, with a correlation coefficient (R^2^) greater than 0.99. The obtained activation energies were 22.81 kJ mol^−1^ for Refractance window (RW) and 31.44 kJ mol^−1^ using conventional hot air drying (CO) while a diffusivity of 2.9 ∗10^−8^ m^2^ s^−1^ for RW and 1.3∗10^−8^ m^2^ s^−1^ for CO were found as well. According to our results, a greater drying efficiency and a less chemical deterioration of the plant sample are obtained by drying with Refractance window.

## 1 Introduction

The objective of the present study was to compare two different drying techniques and demonstrate that RW™, due to its characteristics and performance, is a convenient technique in terms of quality and energy consumption, according to the best data fit to a well-recognized mathematical model. The comparison and contrasting were not done with other dryers such as freeze-drying systems because of the properties of the studied vegetal samples. The studied materials were cellulose and mucilage extracted from the cactus species *Opuntia ficus-indica* (OFI) and *Austrocylindropuntia cylindrica* (CC). These materials were dried by two different systems (conventional oven and Refractance window) in a temperature range between 45 °C and 85 °C, and a comparison was made. The mathematical models tested were Lewis, Henderson Pabis, Page, and the Logarithmic one. These models were statistically compared using the correlation coefficient (R^2^), mean squared error (MSE), and root mean square deviation (RMSD). Additionally, the activation energy (Ea) and diffusivity (D) were calculated; mucilages as well as celluloses were characterized by Fourier Transform Infrared Spectroscopy (FTIR).

*Opuntia ficus-indica* (OFI) is one of the most studied cactus species worldwide. It is cultivated for harvesting nopal and figs, and their crops are periodically pruned while the cladodes are thrown away. Mucilage and cellulose from (OFI) are used in the cosmetic, food, and pharmaceutical industries (Bouaouine et al., 2019; Faccio et al., 2015; Wan et al., 2019). However, these products contain a large amount of water (Bayar et al., 2016) which implies high transport and refrigeration costs for their storage and preservation. Additionally, multiple studies have been carried out showing the potential use of these species in water treatment in recent years (Bouaouine et al., 2019).

On the other hand, *Austrocylindropuntia cylindrica* (CC) is found in the wilderness of the Colombian highlands, but its possible applications in the food, cosmetic, or water purification industries have not been studied so far.

Dehydration is a technique used in the food industry to preserve food and reduce its moisture content. During conventional drying, heat energy is transferred by conduction, convection, or radiation. Depending on the drying system, energy causes transport of water mass from the interior of the sample to the surface where it evaporates (Raghavi et al., 2018). This process leads to a deterioration of the quality of the material due to physical and chemical alterations in its structure (Bonazzi and Dumoulin, 2014). The analysis of the kinetics of the drying process of plant material allows the optimal conditions selection.

Conventional oven or tray drying is the most used method in the industry; however, its low efficiency and the plant samples physicochemical alterations, modify the quality of the product. Refractance window drying, RW™, is classified as a third generation drying technique which transfers energy by conduction, convection, and infrared radiation, through a polyethylene-terephthalate. (Mylar®) semi-transparent sheet. In drying processes, better results are obtained if conductive flux is used instead of radiation flux. The conductive flow is high during the initial drying stage and contributes considerably to the rapid rise in product temperature. As the product temperature increases, conductive flux such as radiation one, decrease. In RW™ drying processes, a lower product temperature is attained due to the formation of an air gap between the product and the formed film because of the product shrinkage by the convective cooling environment of the flowing air over the drying material. This technique allows a reduction in the physical-chemical deterioration of the material and is more energy efficient (Raghavi et al., 2018).

## 2 Materials and methods

*Opuntia ficus-indica* was collected in a private field located in the municipality of Tibasosa (Boyacá, Colombia, 5° 44´23.1´´ North, 72° 58´30.5´´ East) and *Austrocylindropuntia cylindrica* was collected in the Caleras sector of the municipality of Tibasosa (Boyacá 5° 43´04´´ North, 72° 57´15´´ West). *Pan Reac*™ brand ethanol (98%), sodium hydroxide (NaOH), hydrochloric acid (HCl), isopropanol, and hydrogen peroxide (H_2_O_2_) were used throughout the study. First, mucilage and cellulose from the two cacti species were extracted in a home microwave device (Aceb AR HM-0.7 BL) with an adapted reflux system. Then, the materials were dried in a 42L forced air convection laboratory oven (batch process) and in a refractance window (Centricol).

### 2.1 Moisture ratio and drying process

The collected cactus samples were washed, and their spines removed. To obtain the drying curve, the methodology used by (Rosa et al., 2015) was used with some modifications such as scaling temperatures 45 °C, 55 °C, 65 °C, 75 °C, and 85 °C. 2 mm thick pieces weighing 3.0500 ± 0.0500 g were cut and placed into the drying apparatus. Every 15 min, the sample was removed, weighed, and placed back into the drying apparatus. The kinetics study was considered over when the reduction weight of sample was less than 0.0010 g/min. According to the samples origin and the drying process, four different treatments were carried out: *Austrocylindropuntia cylindrica* and *Opuntia ficus-indica* dried in the convection oven (CCO and OFIO), and *Austrocylindropuntia cylindrica*, and *Opuntia ficus-indica* dried in Refractance Window (CCRW and OFIRW, respectively).

### 2.2 Mathematical models for drying kinetics

Equations and the mathematical models are summarized in Table 1. W_t_ is the weight of the moisture sample at time *t*, W_f_ is the weight of the final sample, and W_0_ is the initial weight of the sample.

**Table 1:** Equations used in mathematical modeling.

	Mathematical model	Equation
R^2^	$R^{2}=1-\frac{\sum_{i=1}^{N}{\left(MRpre-MR\;\exp\right)}^{2}}{\sum_{i=1}^{N}{\left(MR\;\exp-\bar{MR}\exp\right)}^{2}}$	Equation (1)
EMC	$EMC=\frac{1}{N}\sum_{i=1}^{N}\left(MR\;\exp-MRpre\right)$	Equation (2)
RECM	$RECM=\frac{1}{N}\sum_{i=1}^{N}{\left(MR\;\exp-MRpre\right)}^{1/2}$	Equation (3)
Moisture Ratio	$MR=\frac{W_{t}-W_{f}}{W_{0}-W_{f}}$	Equation (4)
Arrhenius	$K=Ae^{\left(-\frac{Ea}{RT}\right)}$	Equation (5)
Lewis	${MR=e}^{-kt}$	Equation (6)
Henderson Pabis	${MR=a\;e}^{-kt}$	Equation (7)
Page	${MR=a\;e}^{-kt^{n}}$	Equation (8)
Logarithmic	${MR=a\;e}^{-kt}+c$	Equation (9)
Second law of Fick	$\frac{\delta MR}{\delta t}=Diff\nabla^{2}MR$	Equation (10)
Diffusivity	$\mathrm{Ln}\left(MR\right)=\mathrm{Ln}\left(\frac{8}{\pi^{2}}\right)-\frac{\pi^{2}\;Diff}{4L^{2}}$	Equation (11)

The activation energy was calculated using the Arrhenius equation (Equation 5), where K is the specific constant, A is the pre-exponential factor, EA is the activation energy (kJ/mol), and R is the universal gas constant (8.314 J/mol K).

#### 2.2.1 Lewis model

The Lewis model is a mathematical explanation for drying agricultural products (Equation 6) (Onwude et al., 2016), where MR is the humidity ratio, *k* is the constant of the Arrhenius equation, and *t* is time.

#### 2.2.2 Henderson-Pabis model

The Henderson-Pabis model is related to Fick's second law (Keneni et al., 2019). It has produced a good fit when predicting the drying of the African palm fruit and African dill leaves (Equation 7), where *k* and *a* are the constants of the Arrhenius equation.

#### 2.2.3 Page model

The Page model is an empirical modification of the Lewis model that corrects some of its deficiencies. It is used to describe the drying process of bay leaves, cashews, mango slices, and moringa seeds (Equation 8); *k* and *a* are the constants in the Arrhenius equation, while *n* is the equation's constant.

#### 2.2.4 The logarithmic model

The logarithmic model is presented in Equation 9; where *k* is the constant in the Arrhenius equation, while *a* and *c* are the constants of the logarithmic model equation.

#### 2.2.5 Diffusivity calculation

Moisture transport during the period of downward heat rate can be mathematically analyzed using the diffusion model of Fick's Second Law (Equation 10) (Kouhila et al., 2020). This model can be used to determine diffusivity (Equation 11).

#### 2.2.6 Statistical parameters

To determine the model that explains adequately the behavior of the of the cactus species drying kinetics, several statistical parameters including R2 (Equation 1), MSE (Equation 2), and RMSD (Equation 3), were used. A fitted curve was also applied using the four semi-theoretical mathematical models and the Excel SOLVE tool.

#### 2.2.7 Mucilage extraction

The extraction of mucilage was carried out according to the methodology cited by Felkai-Haddache et al. (2016) with some modifications. The dry material was ground and dispersed in distilled water in a 1:20 ratio. The diluted sample was heated, at 700 W power for 7 min, in a home microwave oven, with a condenser connected to the boiling flask. Afterwards, the sample was centrifuged for 15 min at 4000 rpm. The supernatant was recovered, and the mucilage was precipitated by adding ethanol in a 1:3 ratio and kept overnight at 4 °C. The precipitate was filtered and washed with isopropanol and then dried and powdered. The precipitate from centrifugation was recovered for cellulose extraction.

#### 2.2.8 Cellulose extraction

The cellulose was extracted according to the methodology reported by Singh et al. (2012) with some modifications. The precipitate from the centrifugation of the mucilage extraction process was used to obtain cellulose. 1 M NaOH was added at a ratio of 1:20, for drying and grinding the residues. This was then subjected to microwave radiation for 30 min at a power of 400 W. The solution was then centrifuged at 4000 rpm for 5 min. This process was repeated and washed with distilled water until a neutral pH was reached. Afterwards, 30% H_2_O_2_ was added and subjected to microwave radiation for 15 min at a power of 300 W. After centrifuging at 3000 rpm for 15 min, the material was washed with distilled water and the cellulose obtained was ground and stored for later use.

#### 2.2.9 FTIR analysis

The FTIR analysis was performed using the ATR technique in an IR-Sprint QATAR-S Shimadzu spectrometer, with a wave number range between 400 cm^−1^ and 4000 cm^−1^, and a resolution of 4 cm^−1^ and 32 scans.

## 3 Results and discussion

### 3.1 Effect of temperature

**Figure 1:**
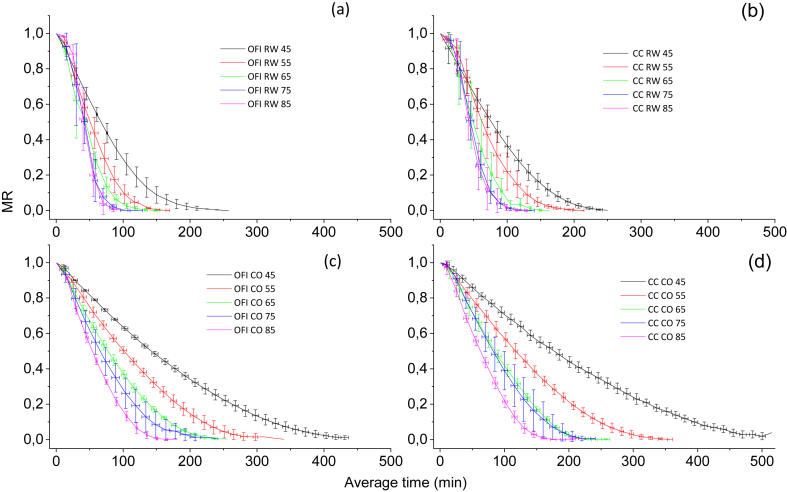
Moisture ratio, MR (grams of dry sample/grams of wet sample) vs drying time (min) at different temperatures (a) Opuntia ficus-indica dried in Refractance Window (RW), (b) *Austrocylindropuntia cylindrica* dried in Refractance Window, (c) Opuntia ficus-indica dried in conventional oven, (d) *Austrocylindropuntia cylindrica* dried in conventional oven.

The drying process of *Opuntia ficus-indica* and *Austrocylindropuntia cylindrica* cladodes was carried out using a convective drying device and a refractance window drying system at different temperatures (45 °C, 55 °C, 65 °C, 75 °C, and 85 °C). Figure 1 shows a decrease in drying time while the temperature was increased in all cases; however, after 65 °C, the graphs overlapped likely due to the hardening of the sample that prevented an increase in the drying rate.

In Figure 1 (a) and (b), which correspond to Refractance window drying, RW™, a notable decrease in drying time is observed compared to Figure 1 (c) and (d), which correspond to drying in a conventional oven. During dehydration, the loss of water causes a hardening phenomenon which increases as the temperature increases. This hardening may be due to an alteration in the physical and chemical structure of the mucilage and cellulose. Differences in drying time between refractance window drying (RW™) and a convective drying device are explained by considering the mechanism of heat transfer effect: while the refractance window drying (RW™) transfers heat energy through the three conduction systems, the conventional hot air-drying transfers heat only by convection. However, no significant differences in drying time were observed in the studied cactus species during dehydration.

These results are corroborated by Keneni et al. (2019), who also reported a hardening of the vegetable sample with the increase in temperature and an overlap in the graphs (Sabarez, 2015). also reported on the efficiency of the refractance window drying (RW™) system. The analysis of the results indicated that there was a structural change in the plant material which varied with temperature. It was also found that the refractance window drying (RW™) system is more efficient since it reduced drying time by up to 50%.

In this process, important sensory qualities of the fresh whole sample, such as color, aroma, and taste, were retained. This is an indicator that the active aromatic and pigment compounds which impart sensory and invaluable nutritional properties, have been preserved throughout the drying process. The product was cool enough for continuous handle while drying. Product temperatures were low and no harsh vacuums destroying cellular structures were present.

### 3.2 Drying rate

**Figure 2:**
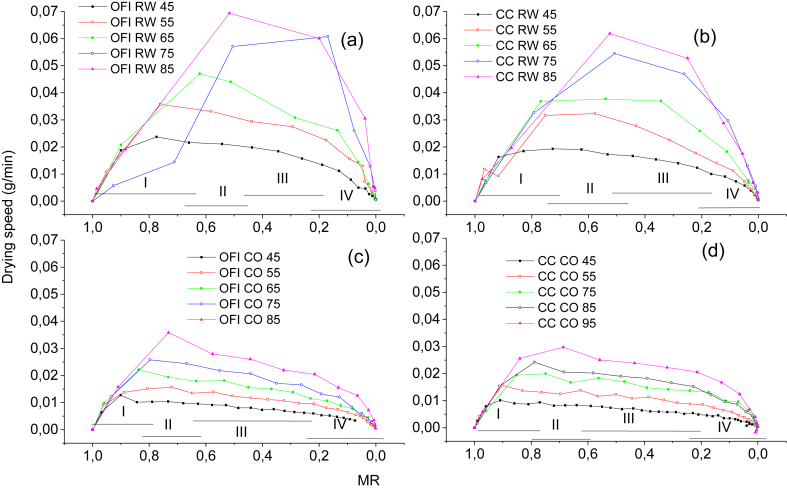
Drying rate (g/min) vs moisture ratio (MR) for (a) Opuntia ficus-indica dried in Refractance window (OFI RW), (b) *Austrocylindropuntia cylindrica* dried in Refractance window (CCRW), (c) Opuntia ficus-indica Oven Dried (OFI CO) and (d) *Austrocylindropuntia*.

In Figure 2 (a) and (b) the drying rate (g/min) of the dehydrated cactus species is compared to the moisture ratio (MR). The drying rate is higher in Refractance window drying, RW™, reaching values of 0.07 g/min, while the drying rate using a conventional oven only reached about 0.035 g/min. The illustrations show four zones: *I* - increase in drying rate, *II* - constant drying rate, *III* - first period of drop-in drying rate, and *IV* - second period of drop-in drying rate. Each graph indicated that the drying rate increased with an increase in temperature.

The drying rate of the cactus using refractance window drying (RW™) was faster and resulted in a shorter drying time as observed in Figure 1. This explains why as the temperature is increased, the drying rate is also increased. In the areas indicated above, the relationship between the change in the structure of the material with respect to the decrease in humidity is inferred. In zone *I*, the material is increasing in temperature, a trend which ends when the equilibrium between the systems and the sample's temperature is reached. It is also the zone with the maximum rate. In zone *II*, a constant period is observed where water migrates by capillarity action to the surface where it evaporates. In zone *III*, the steam is eliminated by diffusion and the drying rate decreases because the free water has been completely removed. The remaining water in the sample is linked by hydrogen bonds to the hydrophilic molecules and the breakdown of the hydrogen bonds requires more energy. In zone *IV*, there is a second drop-in rate which indicates that the remaining water is minimal and is bound inside the sample, which means that heat energy must be increased to extract it. Different studies (Onwude et al., 2016) (Qiu et al., 2019) (Torregroza-Espinosa et al., 2014) reported a similar tendency. These results showed that refractance window drying (RW™) is more efficient than conventional hot air drying. From the above, it can be inferred that the calculation of the (D) values should take place in zones *III* and *IV*.

### 3.3 Mathematical model for drying kinetics

The data obtained from the drying kinetics were normalized as a function of the humidity ratio, and the measurements were made in triplicate. In Table 2, the values obtained for the constants *K*, *a*, and *C* are shown. InFigure 3a and 3b, the experimental data were correlated with four mathematical models. The logarithmic model was better in describing the dehydration of cacti and was confirmed with the average statistical parameters of the temperatures from the non-linear adjustment (Table 3). The values of the constants obtained for each treatment are found in Appendix 1-4.

**Table 2:** Constants obtained for adjusting the mathematical models.

Model	Constants	45 °C	55 °C	65 °C	75 °C	85 °C
Lewis	*K ∗10*^*3*^	*4.56*	*7.14*	*10.05*	*10.30*	*13.99*
Henderson Pabis	*K ∗10*^*3*^	*4.97*	*7.94*	*11.26*	*11.58*	*15.53*
	*a*	*1.08*	*1.13*	*1.13*	*1.16*	*1.15*
Page	*K ∗10*^*3*^	*1.52*	*0.41*	*0.51*	*0.01*	*0.01*
	*a*	*1.42*	*1.79*	*1.86*	*3.02*	*3.04*
Logarithmic	*K∗10*^*3*^	*3.02*	*5.12*	*7.62*	*7.67*	*13.09*
	*a*	*1.34*	*1.34*	*1.39*	*1.41*	*1.31*
	*C*	*-0.29*	*-0.25*	*-0.25*	*-0.27*	*-0.12*

**Figure 3:**
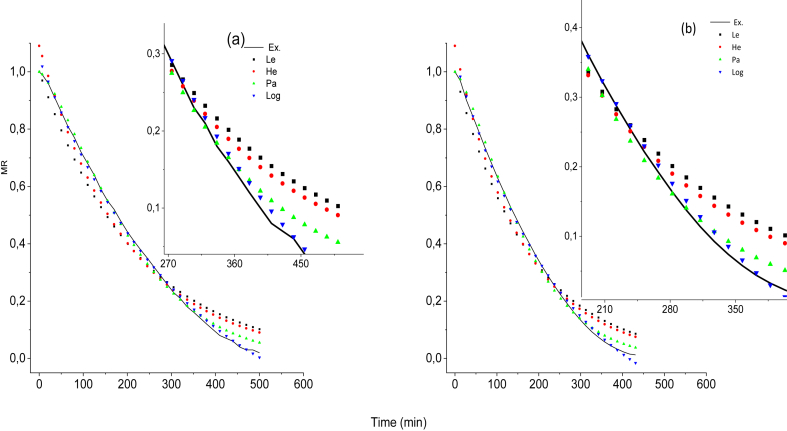
Moisture ratio vs time. Comparison of the mathematical models of Lewis (Le), Henderson Pabis (He), Page (Pa) and Logarithmic (Log) with the experimental kinetics. (a) Hot air-drying system (b) Refractance window drying, RW™, system.

**Table 3:** Statistical parameters obtained for the adjustment of the average mathematical models of the temperatures.

Drying system	Statisticians	Lewis	Henderson	Page	Logarithmic
CC CO	R^2^	0.970 ± 0.0092	0.980 ± 0.0063	0.989 ± 0.0200	0.996 ± 0.0028
	EMC∗10-3	5.9 ± 1.7	5.1 ± 0.2	2.3 ± 4.5	0.7 ± 0.1
	ERMS ∗10-3	76.1 ± 12.4	62.8 ± 10.5	33.0 ± 37.1	23.8 ± 90
OFI RW	R^2^	0.947 ± 0.0265	0.962 ± 0.0207	0.998 ± 0.0013	0.988 ± 0.0086
	EMC∗10-3	12.02 ± 9.75	11.59 ± 6.86	0.27 ± 0.16	2.68 ± 2.15
	ERMS ∗10-3	102.55 ± 43.39	90.74 ± 40.48	13.26 ± 3.80	45.28 ± 25.75

The weighing time contributed to a decrease in the linear adjustment and an increase in statistical error; however, most of the R^2^ values obtained for all of the mathematical models used were greater than 0.95. In Table 3, the Page mathematical model presented a better linear relationship for OFIRW; however, the value of the constant *K* depends on temperature, a behavior that does not occur for this treatment. The constant of the model varies between 1.42 and 3.4, while the constant *a* of the logarithmic model, which must remain constant at different temperatures, fluctuates between 1.31 and 1.41. The statistical parameters obtained for each treatment are found in Appendix 1-4*.*

Similar statistical results reported by (Avhad and Marchetti, 2016) and (Şahin and Öztürk, 2018), where a mathematical model with an R^2^ greater than 0.990 were accepted. It is reported that the maximum variation for the constant *a* of the Page model is 1.00, which supports the rejection proposed in the current study by (Vega Gálvez et al., 2007). In another study (Kouhila et al., 2020), the logarithmic model is reported to describe the drying of mussels and had proved able to best describe the drying behavior of kiwi slides (Azizi et al., 2017). The mathematical adjustment is different according to the studied material. For cacti samples, a similar behavior was observed in terms of the adjustment of the drying kinetic data. After considering the obtained constant *K*, the relative EA was calculated.

### 3.4 Calculation of the relative activation energy

The relative Ea values for CCCO, CCRW, OFICO, and OFIRW were calculated from the slope of Ln (K) (Table 2) vs. the inverse of the drying time. The values obtained were: 31.68 kJmol^-1^ (R^2^ 0.994) for CCCO, 22.81 kJmol^-1^ (R^2^ 0.843) for CCRW, 31.44 kJmol^-1^ (R^2^ 0.989) for OFICO and 24.11 kJmol^-1^ (0.949) OFIRW. Species dried by refractance window drying (RW™) showed a lower Ea compared to those dried in a conventional oven. There is no significant difference in EA between species. On the other hand, the linear statistical parameter R^2^ indicated a good fit of the regressions. The samples dried by refractance window drying (RW™) reported a lower Ea, which explains the efficiency in heat transfer. A lower EA triggers diffusion during drying (Keneni et al., 2019), which would decrease the deterioration of the chemical structure of the material. The Ea depends on the chemical and physical structure of the sample to be dried, but since the cactus species have similar components, they present similar Ea values (Torregroza-Espinosa et al., 2014).

Similar results for other plant species and foods were obtained by (Torres et al., 2012). The reported values indicated that for plant samples, the activation energy is between 15 and 120 kJ/mol. Other studies (Martínez Soto et al., 2010) (Onwude et al., 2016), in which the fluidized bed drying kinetics of *Opuntia ficus indica* was carried out, reported Ea values between 28.21 and 35.85 kJ/mol. These values are comparable to those obtained in the current study using a conventional oven and to the results of Uribe et al. (2022), who reported an Ea of 29.46 kJ/mol at 60 °C, for *Physalis peruviana*. Considering that the value obtained for Ea in the refractance window drying (RW™) process is the lowest, it can be inferred that the use of this technique is more efficient for drying similar materials to cacti.

### 3.5 Effective moisture diffusion

**Figure 4:**
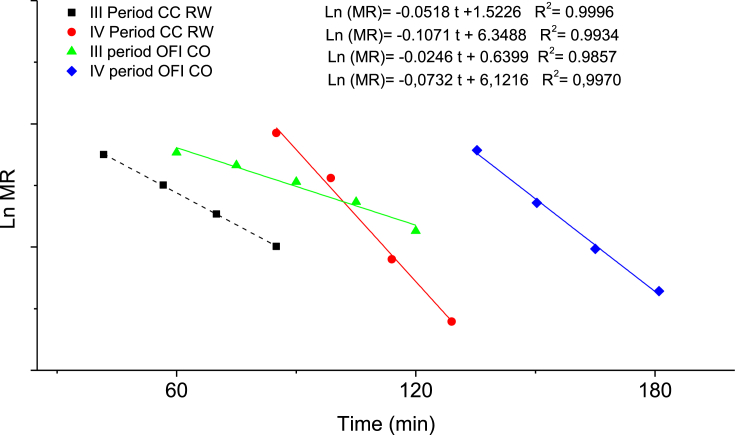
Ln Moisture ratio vs drying time. Slope of period I and II to calculate diffusivity.

**Figure 5:**
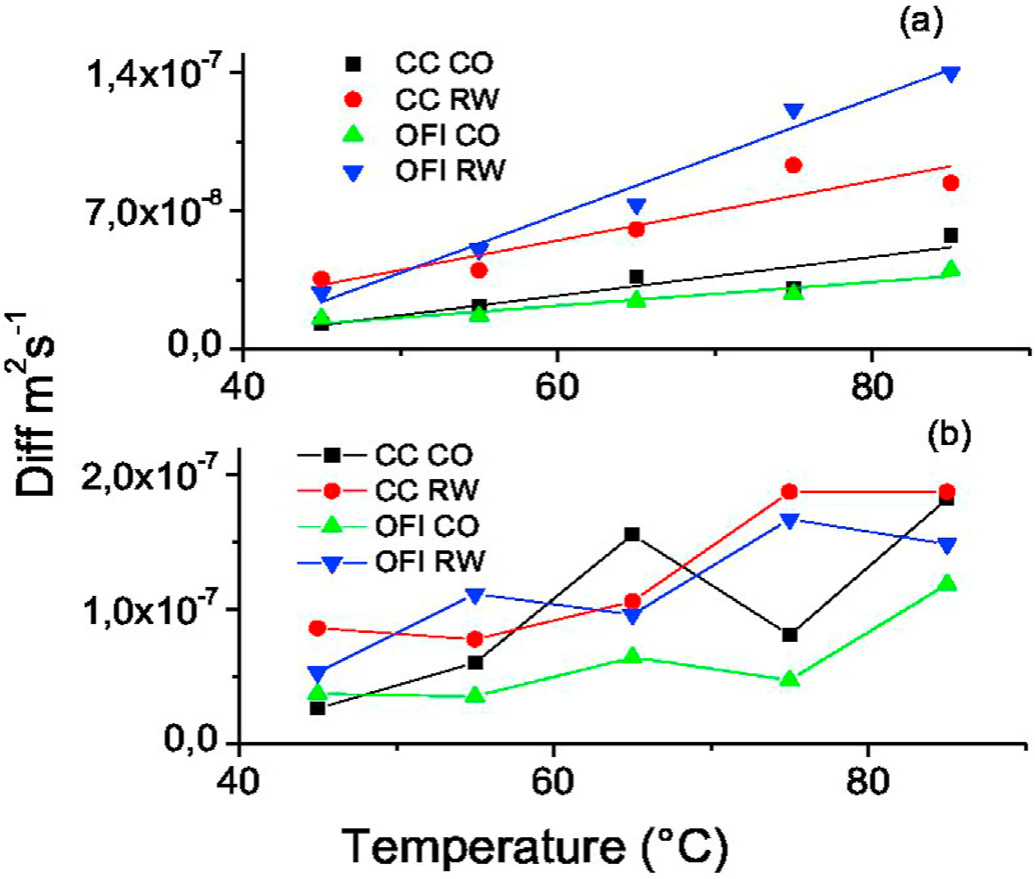
Diffusivity vs Temperature. (a) III period (b) IV period. Opuntia ficus-indica dried in Refractance Window (FV), *Austrocylindropuntia cylindrica* dried in Refractance Window (CV), Opuntia ficus-indica dried in Oven (FH) and *Austrocylindropuntia cylindrica*.

In Figure 4, the relationship between the Ln HR and the drying time is observed for the periods of rate decline, where the effective diffusion was calculated (Appendix 5). The samples dried using refractance window drying (RW™), in general, had a steeper slope, and the slope for the period of decline, zone *IV*, was greater than the period of decline for zone *III*. InFigure 5, the effective value of the moisture diffusion for the decline period *III (a)* and *IV (b)* as a function of the temperature is shown. In Figure 5 *(a)* an increase in (D) was related to the increase in temperature. Additionally, the (D) value of the samples dried using refractance window drying (RW™) was greater than those in conventional oven, with results ranging from 2.9 10 ^−8^ m^2^s^−1^ to 14 10 ^−8^ m^2^s^−1^ and from 1.310 ^−8^ to 5.8 10^−8^ m^2^s^−1^, respectively. It was also observed that the difference between the (D) values in the species dried in a convective drying device is not statistically significant; however, at high temperatures using refractance window drying (RW™), an important difference was noted.

Ea is inversely proportional to (D); as the Ea decreases, the (D) value increases (Potosí-Calvache et al. (2017). Likewise, the temperature affected (D) in a proportional way as seen in Figure 5. The (D) can be understood as the heat gradient that moves in an area per second. Therefore, if this parameter is efficient, the Ea will decrease. The parameters that affect the measurement include dehydration, material composition, and drying temperature. It has been reported (Kartika et al., 2011) (Mannai et al., 2018) that at higher temperatures, the active binding sites of water are destabilized or undergo chemical changes due to molecular vibration. This causes a decrease in the intramolecular forces and the bond breaks resulting in an increase in (D). The refractance window drying (RW™) process increases molecular vibration due to infrared radiation, which causes a decrease in the EA. Drying in a convective drying device resulted in a (D) of 4.26 10^−10^ m^2^ ∗s^−1^ for mango slices, which is lower than the value found in the current study due to the composition of the material.

Previous studies (Mewa et al., 2019) (Potosí-Calvache et al., 2017) showed similar relationships with (D) when the temperature was increased. Diffusivity values increased with increasing temperature and drying air velocity. It is also reported by Azeez et al. (2019) that the (D) interval for plant materials is between 10^−8^ m^2^ s^−1^ and 10–^12^ m^2^ s^−1^, which is similar to the current study. Additionally, changes in (D) were reported as result of alterations in drying conditions. For refractance window drying (RW™), a (D) of 4.26 ∗10^−10^ m^2^ s^−1^ has been reported in mango slices (Salcedo-Mendoza et al., 2016). The values obtained at different temperatures indicated a physical or chemical alteration in the structure of the cacti. This is especially important when considering that cellulose and mucilage have functional groups—active sites for binding with water molecules. On the other hand, higher (D) values with low Ea indicated optimal drying with minimal alterations in the chemical and physical structure of the cacti.

The former results established the evidence-based observation that the thermal efficiency of RW™ drying systems is advisable for vegetal samples; usually it is in the range of 52–77%, barely comparable to drum drying. On the other hand, hot air-drying systems offer only 50% of the efficiency of RW™ dryers. In our laboratory, we are employing mucilages and celluloses from cacti for its functionality and posterior use in flocculating wastewater process. This drying system offers us a more adequate alternative in terms of flocculating efficiency of the obtained products.

On the other hand, water activity (a_w_), one of the most important properties in vegetal materials mainly used in food industry, has been analyzed during this study. The drying technique strongly affected the water activity. By using the RW technique, it is possible to reduce a_w_ below 0.5 in an hour, whereas for tray drying, near 4 h are required to bring a_w_ to 0.5. (Data not included in the present report).

### 3.6 FTIR analysis

**Figure 6:**
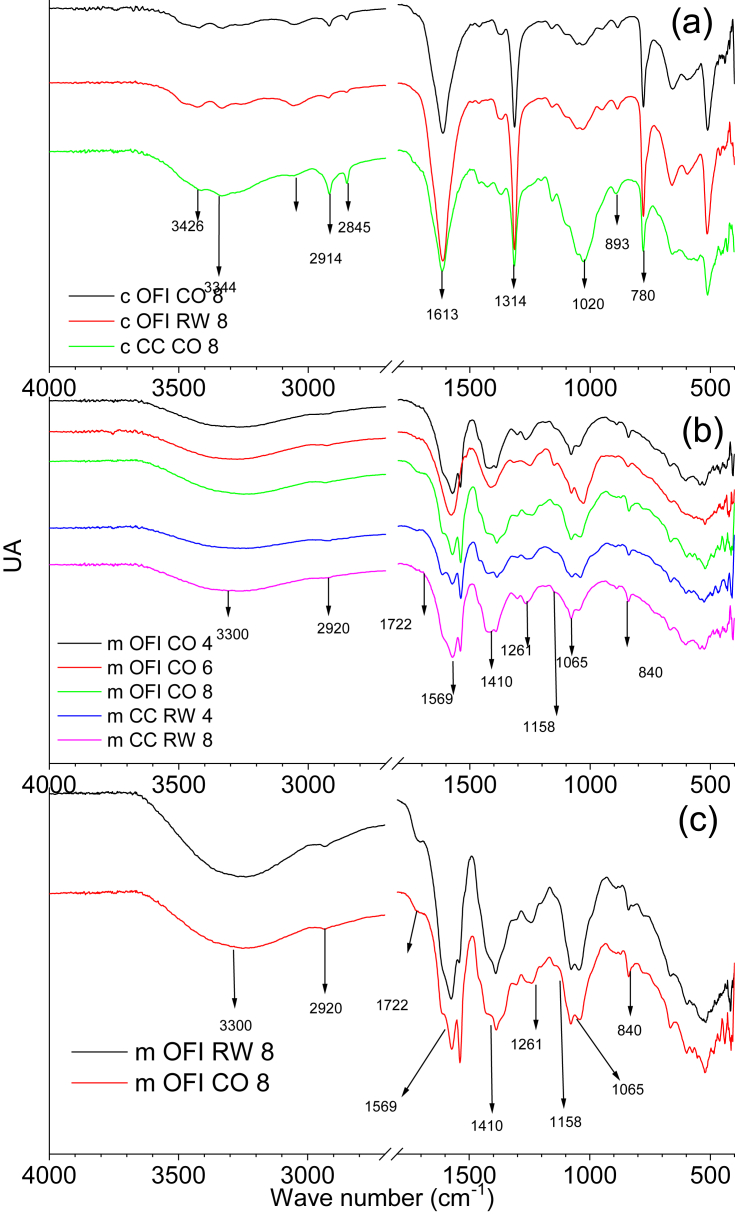
FTIR spectrum of (a) celluloses (b) mucilages (c) mucilages dried at 85 °C.

The FTIR spectra of the cellulose and mucilage showed characteristic signals of their compositional functional groups. In Figure 6 *(a)*, for cellulose, the signals obtained at 3426 cm^−1^ and 3344 cm^−1^ are attributed to the stretching of the OH bond, while the signals between 1914 cm^−1^ and 2845 cm^−1^ are characteristic of the symmetric and asymmetric stretching bond of CH sp^3^ (Bouhdadi et al., 2011). The band at 1613 cm^−1^ is attributed to the carbonyl group (Tamilselvi et al., 2019). The signals observed between 1425 cm^−1^ and 1317 cm^−1^ are from the deformation of the C–H bond. The bands between 1150 cm^−1^ and 1000 cm^−1^ are characteristic of the symmetric and asymmetric deformation of the C–O, C–O–C, and C–O–H bonds. The signal at 893 cm^−1^ is from the β-glucosidic bond, which joins the glucose units that make up cellulose (Vieyra et al., 2015).

The extracted mucilage in Figure 6 *(b)* and *(c)* showed signals at 3300 cm^−1^ of the O–H bonds, while a short signal was observed at 2920 cm^−1^belonging to the C–H sp^3^ stretch. At 1569 cm^−1^, the characteristic free carbonyl signal was observed which is attributed to uronic acid (Otálora et al., 2019). At 1410 cm^−1^, the signal of the symmetric deformation of CH was obtained which is assigned to reducing sugars as well (Faccio et al., 2015). The signal at 1268 cm^−1^ belongs to the acetyl-O glycosidic bond (Zeng et al. (2016). At 1158 cm^−1^, the signal of the C–O–H twisting bond, which is characteristic of arabinose, was observed. At 1065 cm^−1^, several signals were observed. They are the fingerprints of carbohydrates and belong to the twisting of the pyranose ring. At 840 cm^−1^, the characteristic α-glucosidic bond of pectin was observed (Bayar et al., 2017) (Faccio et al., 2015).

No significant differences were observed in the cellulose when it was extracted from cacti dried at different temperatures or by different drying systems; however, it was observed that cellulose extracted from *Opuntia ficus-indica* had a slight signal at 3047 cm^−1^, attributed to the presence of traces of lignin (Agwuncha et al., 2021). The signal present at 1020 cm^−1^ was more intense in cellulose which would indicate the presence of hemicellulose.

For mucilage, no significant changes were observed. The overtones were observed at 1580 cm^−1^ and 1409 cm^−1^ and are attributed to the interactions of the different components of the mucilage. This indicated a variation in the proportions of the sugars present which becomes noticeable at elevated temperatures. The signal at 1065 cm^−1^ and 840 cm^−1^ increased with temperature and was due to an increase in the proportion of polysaccharides.

FTIR analysis indicates that the drying techniques do not significantly alter the structure of 328 the mucilage or cellulose. It was also observed that the temperatures evaluated do not modify 329 the structure; however, there are notable differences related to the species.

## 4 Conclusions

The study reports on the drying comparison of *Opuntia ficus-indica* and *Austrocylindropuntia cylindrica* cacti species by convective drying device dehydration system vs. refractance window. From the analysis of the FTIR spectrograms of mucilage and cellulose, it can be inferred that there was no effect on the structure of the mucilage and cellulose due to the action of the drying system or the temperature. The RW system reduced the dehydration time by 50%, decreased the activation energy by up to 8.63 kJ/mol and doubled the diffusivity. Taking these results into account, the window drying system reduced the drying time without modifying the mucilage or cellulose chemical structure, making it a suitable system for the dehydration of cacti.

## Declaration

### Author contribution statement

Oscar J. Medina: Contributed reagents, materials, analysis tools or data; Wrote the paper.

William Patarroyo. Performed the experiments; Wrote the paper.

Lucia M. Moreno: Conceived and designed the experiments; Analyzed and interpreted the data.

### Funding statement

Lucia Marlen Moreno was supported by Departamento Administrativo de Ciencia, Tecnología e Innovación (COLCIENCIAS) (66016).

This work was supported by Universidad Pedagógica y Tecnológica de Colombia (SGI 2632).

### Data availability statement

Data will be made available on request.

### Declaration of interest's statement

The authors declare no conflict of interest.

### Additional information

No additional information is available for this paper.

## Appendix A Supplementary data

Suplementary Material - Appendices Suplementary Material - Appendices
