# Corrigendum to “Current trends in cacti drying processes and their effects on cellulose and mucilage from two Colombian cactus species” [Heliyon Volume **8**, Issue 12, December 2022, article e12618]

**DOI:** 10.1016/j.heliyon.2025.e43309

**Published:** 2025-05-03

**Authors:** Oscar J. Medina, William Patarroyo, Lucia M. Moreno

**Affiliations:** Grupo de Investigación en Química y Tecnología de Alimentos, Universidad Pedagógica y Tecnológica de Colombia, Colombia

In the original published version of this article, a reference was missing from the reference list but used as a citation in the main text:

*To obtain the drying curve, the methodology used by* ***(Rosa et al., 2015)*** *was used with some modifications such as scaling temperatures 45 °C, 55 °C, 65 °C, 75 °C, and 85 °C. 2 mm thick pieces weighing 3.0500 ± 0.0500 g were cut and placed into the drying apparatus.*

The citation should refer to the reference below:

D. P. Rosa, D. Cantú-Lozano, G. Luna-Solano, T. C. Polachini, and J. Telis-Romero, “MATHEMATICAL MODELING OF ORANGE SEED DRYING KINETICS,” *Ciência e Agrotecnologia*, vol. 39, no. 3, 2015, doi: 10.1590/s1413-70542015000300011.

The authors apologize for the errors.

## Declaration of competing interest

The authors declare no conflict of interest.

