# Supplementary material for: Current trends in cacti drying processes and their effects on cellulose and mucilage from two Colombian cactus species
Source: Heliyon. 2022 Dec 24;8(12):e12618. doi: 10.1016/j.heliyon.2022.e12618 (PMC9816971; doi:10.1016/j.heliyon.2022.e12618)
Supplement: Suplementary Material - Appendices [file mmc1.docx]

# Appendices

Appendix 1. *Value of the constants obtained by fitting the Lewis mathematical model.*

| **Lewis** | | | | | |
| --- | --- | --- | --- | --- | --- |
| CC CO | 45 | 55 | 65 | 75 | 85 |
| K | 0,005 | 0,007 | 0,010 | 0,010 | 0,014 |
| R^2^ | 0,985 | 0,975 | 0,964 | 0,967 | 0,963 |
| X^2^ | 0,003 | 0,005 | 0,007 | 0,007 | 0,007 |
| ERMS | 0,057 | 0,071 | 0,084 | 0,086 | 0,083 |
| CC RW | 45 | 55 | 65 | 75 | 85 |
| K | 0,011 | 0,014 | 0,018 | 0,020 | 0,021 |
| R^2^ | 0,982 | 0,959 | 0,954 | 0,916 | 0,925 |
| X^2^ | 0,004 | 0,008 | 0,009 | 0,018 | 0,017 |
| ERMS | 0,060 | 0,090 | 0,094 | 0,132 | 0,129 |
| OFI CO | 45 | 55 | 65 | 75 | 85 |
| K | 0,006 | 0,008 | 0,011 | 0,013 | 0,016 |
| R^2^ | 0,985 | 0,979 | 0,980 | 0,981 | 0,969 |
| X^2^ | 0,003 | 0,004 | 0,004 | 0,004 | 0,006 |
| ERMS | 0,056 | 0,063 | 0,063 | 0,059 | 0,077 |
| OFI RW | 45 | 55 | 65 | 75 | 85 |
| K | 0,013 | 0,017 | 0,021 | 0,022 | 0,022 |
| R^2^ | 0,980 | 0,965 | 0,965 | 0,919 | 0,898 |
| X^2^ | 0,003 | 0,007 | 0,006 | 0,017 | 0,027 |
| ERMS | 0,058 | 0,083 | 0,078 | 0,131 | 0,163 |

| Appendix 2. Value of the constants obtained by fitting the Henderson Pabis mathematical model. Henderson Pabis Equation | | | | | |
| --- | --- | --- | --- | --- | --- |
| CC CO | 45 | 55 | 65 | 75 | 85 |
| K | 0,005 | 0,008 | 0,011 | 0,012 | 0,016 |
| a | 1,091 | 1,117 | 1,132 | 1,133 | 1,127 |
| R^2^ | 0,990 | 0,984 | 0,976 | 0,979 | 0,975 |
| X^2^ | 0,005 | 0,005 | 0,006 | 0,005 | 0,005 |
| ERMS | 0,047 | 0,058 | 0,070 | 0,070 | 0,070 |
| CC RW | 45 | 55 | 65 | 75 | 85 |
| K | 0,012 | 0,015 | 0,020 | 0,022 | 0,023 |
| a | 1,085 | 1,135 | 1,133 | 1,160 | 1,150 |
| R^2^ | 0,987 | 0,973 | 0,968 | 0,937 | 0,944 |
| X^2^ | 0,005 | 0,009 | 0,008 | 0,014 | 0,013 |
| ERMS | 0,052 | 0,075 | 0,080 | 0,117 | 0,114 |
| OFI CO | 45 | 55 | 65 | 75 | 85 |
| K | 0,006 | 0,009 | 0,012 | 0,014 | 0,017 |
| a | 1,090 | 1,098 | 1,097 | 1,102 | 1,111 |
| R^2^ | 0,990 | 0,986 | 0,986 | 0,987 | 0,978 |
| X^2^ | 0,005 | 0,005 | 0,004 | 0,003 | 0,004 |
| ERMS | 0,046 | 0,053 | 0,053 | 0,050 | 0,066 |
| OFI RW | 45 | 55 | 65 | 75 | 85 |
| K | 0,014 | 0,019 | 0,023 | 0,025 | 0,025 |
| a | 1,093 | 1,120 | 1,102 | 1,139 | 1,161 |
| R^2^ | 0,986 | 0,975 | 0,973 | 0,935 | 0,920 |
| X^2^ | 0,005 | 0,008 | 0,007 | 0,016 | 0,022 |
| ERMS | 0,049 | 0,070 | 0,069 | 0,119 | 0,147 |

| Appendix 3. Value of the constants obtained by fitting the Page mathematical model. Page Equation | | | | | |
| --- | --- | --- | --- | --- | --- |
| CC CO | 45 | 55 | 65 | 75 | 85 |
| K | 0,001 | 0,000 | 0,000 | 0,000 | 0,001 |
| n | 1,347 | 1,637 | 1,637 | 3,101 | 1,701 |
| R^2^ | 0,999 | 0,998 | 0,998 | 0,954 | 0,999 |
| X^2^ | 0,000 | 0,000 | 0,000 | 0,010 | 0,000 |
| ERMS | 0,012 | 0,020 | 0,020 | 0,099 | 0,014 |
| CC RW | 45 | 55 | 65 | 75 | 85 |
| K | 0,002 | 0,000 | 0,001 | 0,000 | 0,000 |
| n | 1,423 | 1,791 | 1,857 | 3,017 | 3,044 |
| R^2^ | 0,999 | 1,000 | 1,000 | 0,997 | 0,997 |
| X^2^ | 0,001 | 0,000 | 0,000 | 0,001 | 0,001 |
| ERMS | 0,018 | 0,010 | 0,010 | 0,028 | 0,030 |
| OFI CO | 45 | 55 | 65 | 75 | 85 |
| K | 0,001 | 0,001 | 0,001 | 0,002 | 0,001 |
| n | 1,371 | 1,449 | 1,456 | 1,464 | 1,595 |
| R^2^ | 0,998 | 0,999 | 0,999 | 1,000 | 0,999 |
| X^2^ | 0,001 | 0,001 | 0,000 | 0,000 | 0,000 |
| ERMS | 0,018 | 0,017 | 0,016 | 0,009 | 0,015 |
| OFI RW | 45 | 55 | 65 | 75 | 85 |
| K | 0,002 | 0,001 | 0,001 | 0,000 | 0,000 |
| n | 1,442 | 1,687 | 1,676 | 2,439 | 3,101 |
| R^2^ | 0,999 | 1,000 | 0,998 | 0,999 | 1,000 |
| X^2^ | 0,000 | 0,000 | 0,001 | 0,000 | 0,000 |
| ERMS | 0,011 | 0,009 | 0,019 | 0,015 | 0,012 |

Appendix 4*.* *Value of the constants obtained by fitting the Page mathematical model.* Logarithmic Equation

| CC CO | 45 | 55 | 65 | 75 | 85 |
| --- | --- | --- | --- | --- | --- |
| K | 0,003 | 0,005 | 0,008 | 0,008 | 0,013 |
| a | 1,338 | 1,345 | 1,389 | 1,413 | 1,314 |
| C | -0,292 | -0,254 | -0,247 | -0,271 | -0,120 |
| R^2^ | 0,999 | 0,998 | 0,997 | 0,997 | 0,992 |
| X^2^ | 0,000 | 0,001 | 0,001 | 0,001 | 0,002 |
| ERMS | 0,013 | 0,018 | 0,025 | 0,026 | 0,037 |
| CC RW | 45 | 55 | 65 | 75 | 85 |
| K | 0,008 | 0,012 | 0,019 | 0,023 | 0,020 |
| a | 1,260 | 1,331 | 1,404 | 1,623 | 1,497 |
| C | -0,188 | -0,135 | -0,102 | -0,127 | -0,168 |
| R^2^ | 0,998 | 0,990 | 0,993 | 0,985 | 0,975 |
| X^2^ | 0,001 | 0,003 | 0,001 | 0,002 | 0,004 |
| ERMS | 0,019 | 0,043 | 0,034 | 0,052 | 0,069 |
| OFI CO | 45 | 55 | 65 | 75 | 85 |
| K | 0,004 | 0,006 | 0,008 | 0,012 | 0,015 |
| a | 1,306 | 1,261 | 1,277 | 1,247 | 1,340 |
| C | -0,260 | -0,182 | -0,182 | -0,094 | -0,128 |
| R^2^ | 0,999 | 0,997 | 0,998 | 0,997 | 0,997 |
| X^2^ | 0,000 | 0,001 | 0,000 | 0,001 | 0,000 |
| ERMS | 0,011 | 0,022 | 0,018 | 0,021 | 0,021 |
| OFI RW | 45 | 55 | 65 | 75 | 85 |
| K | 0,012 | 0,016 | 0,023 | 0,025 | 0,016 |
| a | 1,226 | 1,351 | 1,355 | 1,609 | 1,792 |
| C | -0,093 | -0,130 | -0,064 | -0,142 | -0,472 |
| R^2^ | 0,996 | 0,996 | 0,991 | 0,978 | 0,972 |
| X^2^ | 0,001 | 0,001 | 0,002 | 0,004 | 0,006 |
| ERMS | 0,023 | 0,027 | 0,034 | 0,061 | 0,083 |
